# *Kingella kingae* infections in children

**DOI:** 10.1186/s12879-015-0986-9

**Published:** 2015-07-07

**Authors:** Nicola Principi, Susanna Esposito

**Affiliations:** Pediatric Highly Intensive Care Unit, Department of Pathophysiology and Transplantation, Università degli Studi di Milano, Fondazione IRCCS Ca’ Granda Ospedale Maggiore Policlinico, Milan, Italy

**Keywords:** Emerging infection, Children, *Kingella kingae*, Pediatric infectious disease

## Abstract

**Background:**

Improvements in culture techniques and molecular detection methods have led to findings indicating that, particularly in infants and young children, *Kingella kingae* is a significantly more important pathogen than previously thought. However, despite this, the pediatric community is still largely unaware of the existence of this organism. The aim of this review is therefore to summarise current knowledge of the epidemiology, transmission, clinical presentation, diagnosis and treatment of *K. kingae* infections in children.

**Discussion:**

*K. kingae* is a common coloniser of the oropharynx, can be transmitted from child to child, and can cause outbreaks of infection. Invasive infections almost exclusively occur in children aged between six months and four years of age, and involve mainly joints and bone, less frequently the endocardium, and very rarely other localisations. With the exception of bacteremia and endocarditis, which can be followed by severe complications, the diseases due to *K. kingae* are usually accompanied by mild to moderate clinical signs and symptoms, and only slightly altered laboratory data. Moreover, they generally respond to widely used antibiotics, although resistant strains are reported. However, the mild symptoms and limited increase in the levels of acute phase reactants create problems because *K. kingae* disease may be confused with other clinical conditions that have a similar clinical picture.

**Conclusions:**

Although *K. kingae* was identified more than 50 years ago, it is poorly known by pediatricians and is not systematically sought in laboratories. Education is therefore necessary in order to reduce the risk of outbreaks, permit the early identification of *K. kingae* infections, and allow the prompt prescription of adequate therapeutic regimens capable of avoiding the risk of a negative evolution in those cases in which this elusive pathogen can cause significant clinical problems.

## Review

### Background

For most of the three decades following its first description in 1960*, Kingella kingae* was considered as a rare cause of human disease that was only infrequently isolated from patients with skeletal infections and endocarditis [1]. However, since the early 1990s, improvements in culture techniques and molecular detection methods, together with the increasing familiarity of clinical microbiology laboratories with its identification, have shown that it is significantly more important than previously thought, particularly in infants and young children [1]. It is now recognised as a frequent cause of bacteremia and osteoarticular infections in children aged less than four years [2, 3], and has been associated with some cases of rapidly progressive, complicated endocarditis [4] and, albeit rarely, cases of pneumonia, meningitis, ocular infections, pericarditis and peritonitis [5–9]. There are also reports of outbreaks of *K. kingae* infections in day care facilities [1].

However, despite this, the pediatric community is still largely unaware of the existence of this organism. The aim of this review is therefore to summarise current knowledge of the epidemiology, transmission, clinical presentation, diagnosis and treatment of *K. kingae* infections in children. PubMed was used to search for all of the studies published over the last 15 years using the key words: “*Kingella kingae*” and “children” or “pediatric”. More than 200 articles were found, but only those published in English or providing evidence-based data were included in the evaluation.

### Discussion

#### The pathogen and its identification

*K. kingae* is a facultative anaerobic, β-hemolytic, Gram-negative organism [1] that is difficult to identify in routine solid cultures of blood or body fluids such as synovial fluid or bone exudates as it is isolated in less than 10 % of truly positive cases. However, significantly more cases have been reported when exudates are inoculated into aerobic blood culture vials, particularly when the positive samples are sub-cultured on a blood-agar plate of trypticase soy agar with 5 % sheep blood hemoglobin or chocolate agar. This need for specific culture techniques explains why the etiology of a number of invasive diseases due to *K. kingae* (particularly septic arthritis and osteomyelitis in young children) was not initially identified, thus leading to the definition of “culture-negative bone infections of unknown origin” [1].

It is even more difficult to identify *K. kingae* in cultured pharyngeal samples because of its relatively slow growth and the high density of resident bacterial flora, although this can be overcome by using a selective medium consisting of blood-agar with added vancomycin to inhibit the growth of competing flora [1].

The best means of detecting *K. kingae* are the recently developed nucleic acid amplification assays because they are not only significantly more sensitive than culture, but also reduce the time needed for bacterial identification from 3–4 days to a few hours. They also make it possible to use pharyngeal secretions to diagnose invasive *K. kingae* invasive infections, as described by Ceroni *et al.* who used a polymerase chain reaction (PCR) to identify the etiology of a *K. kingae* osteoarticular infection from pharyngeal swabs [10]. They found that the method was 100 % sensitive and 90.5 % specific, thus making a negative swab sufficient to rule out *K. kingae* infection and prompt more invasive diagnostic measures. However, *K. kingae* detection in the oropharynx of a child with an osteoarticular infection is not an irrefutable proof of the etiology of the disease because the carriage rate of the organism among pediatric patients is around 10-12 % [1].

Furthermore, the molecular typing of isolates has revealed genomic heterogeneity in *K. kingae* species, and made it possible to study the possible association between the genetic characteristics of the different strains and their tendency to cause invasive disease, as well as the relationships between antibiotic resistance and genotype distribution. One of the most widely used molecular techniques is the amplification and sequencing of polymorphisms of the *rtx*A gene, which is involved in the production of a protein belonging to the RTX toxin family that has been associated with the virulence of *K. kingae* insofar as disruption of the RTX locus leads to the loss of cytotoxicity for respiratory epithelial, synovial, and macrophage cell lines [11]. Furthermore, when Chang *et al.* [12] infected rats with *K. kingae* strain PYKK081 and its isogenic *rtx*A-deficient strain KKNB100, they found that PYKK081 causing a fatal illness characterised by rapid weight loss, bacteremia, the formation of necrotic abdominal lesions, and significant histopathology in thymus, spleen and bone marrow, whereas KKNB100 was less toxic and did not induce weight loss, bacteremia or histopathological changes. In addition, the animals injected with KKNB100 had significantly high circulating white blood cell (WBC) counts, whereas the counts of the rats injected with PYKK081 were similar to those recorded in the uninfected controls [12].

The use of pulsed-field electrophoresis (PFGE) has made it possible to establish that a number of the *K. kingae* clones that are relatively frequently isolated asymptomatic carriers (A, C, G, J, M, R, T and U) play a marginal role in determining invasive infections, whereas clones B, H, K, N and P are significantly more frequently associated with the development of disease [13]. This suggests that the more invasive strains may be more rapidly cleared from the respiratory tract and that their persistence may require a different biological specialisation. It was also found that each of the virulent clones may be responsible for a well-defined disease as clone K was significantly associated with bacteremia, clone N with skeletal system infections, and clone P with bacterial endocarditis [13].

It was initially shown that *K. kingae* isolates were almost always susceptible to most of the antibiotics that are routinely administered to children with suspected bacteremia or skeletal system infections, including penicillin, ampicillin, second- and third-generation cephalosporins, macrolides, rifampin, co-trimoxazole, ciprofloxacin, tetracycline and chloramphenicol, whereas oxacillin, clindamycin and daptomycin were not very effective, and trimethoprim and glycopeptide antibiotics had no activity at all [14]. However, resistance of *K. kingae* to β-lactam antibiotics has more recently been repeatedly reported, although the rates vary widely from one country to another: Basmaci *et al.* screened 778 isolates from Iceland, the USA, France, Israel, Spain and Canada for β-lactamase production, and found that the French, Spanish and Canadian isolates were negative, whereas 28.6 % of the Icelandic, 25.0 % of the American, and 11 % of the Israeli isolates were positive [15]. The distribution of β-lactam antibiotic resistance among invasive and carried organisms has not yet been precisely defined, but Yagupsky *et al.* found enzyme production in 1.1 % of the invasive organisms and 15.4 % of the carried organisms detected in a sample of 619 *K. kingae* isolates from Israel [16]. This finding seemed to be confirmed by the PFGE studies of the genotypic clonality of the strains because β-lactamase production was limited to only four of the 73 identified clones (33 in invasive and 56 in carriage isolates), and these four were common among the carried strains but rare among the invasive strains [16]. However, Basmaci *et al.* found that all of their American and Icelandic β-lactamase-positive isolates belonged to the main international invasive PFGE clone K/MLST ST-6, and were different from the four genetically unrelated Israeli β-lactamase-producing clones [15]. The presence of the enzyme in the isolates belonging to the major worldwide invasive *K. kingae* clone highlights the possible spread of β-lactam resistance, and emphasises the importance of routinely testing all *K. kingae* clinical isolates for β-lactamase production.

#### Carriage and transmission

Asymptomatic colonisation of the upper respiratory tract by *K. kingae* is extremely common in children, who acquire the infectious agent after six months of life [1]; subsequently, the incidence of colonization tends to decrease to 10-12 % until the end of the second year before gradually declining to very low levels in older children and adults (Table 1). This suggests that the disappearance of vertically transmitted immunity and the greater socialisation of children aged more than six months increase the risk of colonisation, whereas progressive immunological maturation and/or accumulative familiarity with *K. kingae* antigens as a result of mucosal colonisation lead to the acquisition of sufficient immunity to eradicate the organism from the pharynx in older people. This seems to be confirmed by the results of a study designed to evaluate the dynamics of *K. kingae* antibody levels in childhood, which showed that mean IgG level is high at the age of two months, gradually decreases until the age of 6–7 months (the time of lowest concentrations), remains low until the age of 18 months, and subsequently increases [17].

**Table 1:** *Kingella kingae* carriage

Characteristic	Finding
Main interested population	Young children attending day-care
Seasonality	Mainly detected in late winter and spring
Risk factor	Great socialisation
Protective factor	Immunological maturation
Site of colonisation	Oropharynx
Role of colonisation	Prerequisite for the development of disease

Carriage may be continuous for weeks or months, or intermittent as shown by the findings of an 11-month longitudinal study of children aged 19–48 months old attending a day care centre in Israel [18]: about 73 % carried the organism at least once and half carried it for at least two months during the follow-up period. Moreover, colonisation was greatest during late winter and spring, and characteristically involved oropharynx because *K. kingae* was hardly ever found in the nasopharynx [18].

Colonised children are the main cause of the spread of the pathogen. This was suggested by the fact that colonisation rates are significantly higher in children attending day care centres than in the general population of the same age, and was confirmed by a comparative genetic analysis of cultured *K. kingae* isolated from colonised children and their siblings and playmates that clearly revealed the indistinguishable genotype profiles of the strains identified in both groups [19]. Moreover, although it is asymptomatic in the great majority of children, colonisation is a prerequisite for the development of disease, and genotypically identical isolates have been recovered from the pharynx and bloodstream of patients with invasive *K. kingae* infections [20]. On the basis of these findings, it is not surprising that a number of outbreaks of *K. kingae* disease have been associated with day care centres [3, 21, 22]: in all of these cases, unusually high *K. kingae* colonisation rates were found among the asymptomatic attendees, and all the pharyngeal isolates detected in the classrooms were genetically identical to those identified in patients [9].

#### Clinical manifestations

The pathogenesis of invasive *K. kingae* infection has not yet been defined, but some data seem to indicate that its penetration is favoured by a concomitant viral infection capable of damaging oropharyngeal mucosa. Stomatitis and symptoms of respiratory infection are common in patients with *K. ingaek* disease [23]. The pathogen was isolated from the blood of four out of 29 patients with primary herpetic gingivostomatitis [24], and it has been suggested that, after penetration, it can progress to the lower respiratory tract or invade the bloodstream and subsequently reach the skeletal system, heart or other parts of the body [18].

Invasive infectious *K. kingae* disease generally develops in younger and otherwise healthy children, and its age distribution is similar to that of carriage. Almost 90 % of the reported cases have occurred in children aged <5 years, and 60 % in those aged <2 years [18]. As in the case of invasive *Streptococcus pneumoniae* and *Neisseria meningitidis* infections [25, 26], invasive *K. kingae* infections are more common in males [2].

In most cases, *K. kingae* bacteremia is detected concomitantly with infections of the skeletal, cardiovascular, respiratory or central nervous systems; however, occult bacteremia has been diagnosed in a number of cases, some of which were characterised by a maculopapular rash resembling those seen in patients with disseminated meningococcal disease [27]. The fact that *K. kingae* is identified in the blood of very few patients with ascertained invasive infection even when sensitive and specific molecular methods are used suggests that the duration of bacteremia is usually short [27].

The most frequent clinical manifestation of invasive *K. kingae* infection are osteoarticular infections [1], and *K. kingae* is the most common cause of such infections in children aged between six months and four years [28]: one recent molecular study by Ceroni *et al.* found that 82 % of the joint or bone aspirates of children aged <4 years with osteoarticular infection were positive [29]. Unlike the joint or bone infections due to other bacterial pathogens (mainly *Staphylococcus aureus*), which usually have a severe clinical picture, osteoarticular *K. kingae* infections are generally characterised by mild to moderate clinical and radiological manifestations, and a limited biological inflammatory response [1, 2, 28, 29]. Ceroni *et al.* observed that fewer than 15 % of their children were febrile, 39 % had normal C-reactive protein (CRP) levels, and only 9 % had high WBC counts [29]; Dubnoz-Raz *et al.* found fever in 25 % of their patients, a WBC count of >15,000/mm^3^ in only about 50 %, and CRP levels within the normal range in 22 % [2]. These findings led to the development of an algorithm for the reliable prediction of the *K. kingae* etiology of an osteoarticular disease in younger children that includes a body temperature of <38 °C, CRP levels of <55 mg/L, a WBC count of <14,000/mm^3^, and bands of <150/mm^3^ [30]. However, the possibility to predict osteoarticular *K. kingae* infections on the bases of mild clinical symptoms and acute phase reactants is disputed and still controversial.

Septic arthritis is mainly diagnosed in the large weight-bearing joints, with synovial fluid WBC counts of <50,000/mm^3^ in about 25 % of patients, thus underlining their poor inflammatory response [18]. However, the limited clinical evidence of septic arthritis due to *K. kingae* can prevent a prompt diagnosis in certain cases, thus leading to an increased risk of greater morbidity due to delayed treatment. *K. kingae* septic arthritis of the hip is a good example because it may be impossible to differentiate it from transient synovitis on purely clinical grounds. In an attempt to improve the identification of septic arthritis of the hip, *Kocher et al.* developed a specific algorithm based on clinical variables (body temperature upon admission and a refusal to bear weight) and laboratory data (WBC counts and the erythrocyte sedimentation rate [ESR]) that allows the probable exclusion of septic arthritis when WBC counts and the ESR are within the normal range, there is little or no fever, and there are no demonstrable weight bearing problems [31]. However, although the algorithm may be valid in the case of septic arthritis due to other bacteria [32], it cannot be used in the case of *K. kingae* disease in which all or some of the variables overlap those found in transient synovitis and variation between laboratories may influence the results [33]. Osteomyelitis mainly involves the long bones but also frequently affects bones that are not usually infected by other organisms, such as the calcaneus, talus, sternum and clavicle [34–36].

Spondylodiscitis is another quite frequent clinical manifestation of *K. kingae* infection [1]. Like osteomyelitis and septic arthritis, spondylodiscitis due to *K. kingae* is mainly diagnosed in children aged between six months and four years [37]. The lumbar intervertebral spaces are most frequently affected by mild to moderate signs and symptoms.

Endocarditis is the most severe manifestation of *K. kingae* infection. It usually occurs in children who are a little older than those suffering from osteoarticular infections and, unlike osteomyelitis and septic arthritis, is accompanied by a high fever and a significant increase in the levels of acute phase reactants [4]. As of July 2014, a total of 42 patients with *K. kingae* endocarditis had been described in the literature [4], at least 20 % of whom were aged >4 years. At the time of presentation, most of the patients had a body temperature of >39 °C, and mean ESR and CRP levels were respectively 60.4 mm/h and 12.5 mg/dL. Valvular disease was relatively frequent, particularly in the older children, although only a minority had previously been diagnosed as having congenital heart disease. However, the most serious problem associated with *K. kingae* endocarditis is the emergence of embolic complications that can lead to severe neurological consequences, which have occurred in 13 of the 42 described cases: the most frequent are stroke (77 %) and meningitis (46 %), which have led to the death of four patients (10 %) [4]. Other complications of *K. kingae* endocarditis include valvular insufficiency, cardiogenic shock, pulmonary infarction and paravalvular abscesses [4].

Other infections such those involving the lower respiratory tract, the central nervous system and the eye are rare [5–8]. In particular, meningitis seems to be different from the more common *K. kingae* infections because instead of being diagnosed in younger children, it has mainly occurred in adolescents [5, 38].

Table 2 summarizes the main clinical presentations.

**Table 2:** Main clinical presentations of *Kingella kingae* infection

Clinical presentation	Characteristic
Occult bacteremia	Mainly in children aged <5 years, more common in males, sometimes with a maculopapular rash
Osteoarticular infection	Generally characterised by mild to moderate clinical and radiological manifestations, limited inflammatory response, mainly in the long bones or in bones that are not infected by other organisms (*i.e.*, calcaneus, talus, sternum, and clavicle)
Septic arthritis	Mainly in the large weight-hearing joints, limited clinical evidence, limited inflammatory response
Spondylodiscitis	Mainly in children aged <5 years,, mild to moderate signs in the lumbar intervertebral spaces
Endocarditis	Most severe manifestation of *K. kingae* infection, characterised by fever >39 °C and increased inflammatory markers, possibility of embolic complications with severe neurologic consequences as well as valvular insufficiency, cardiogenic shock, pulmonary infarction and paravalvular abscesses
Meningitis	Mainly in adolescents
Lower respiratory tract infection	Rare

#### Prognosis and treatment

Most *K. kingae* infections are susceptible to the majority of the oral and parenteral antibiotics usually prescribed for young febrile children and, although some invasive β-lactamase-producing strains are resistant to β-lactam antibiotics and require appropriate monitoring, they do not seem to condition the final prognosis of *K. kingae* invasive diseases. Consequently, although no comparative controlled study has been published and it is not possible to establish the best antibiotic approach, most invasive diseases due to *K. kingae* have a benign clinical course when adequately diagnosed. However, diagnostic delays and hospital admission are very common. Skeletal infections generally recover without sequelae provided that they are immediately and appropriately treated including spondylodiscitis, although persistent narrowing of the intervertebral space may remain [39]. The only exception is endocarditis, mainly because of its embolic complications, the most common of which is cerebral infarction [4]. Therefore, when the diagnosis of *K. kingae* endocarditis is suspected or ascertained, health authorities recommend the prompt use of appropriate laboratory methods in order to get information concerning antimicrobial susceptibility as quickly as possible [40]. According to the clinical presentation, the first line therapy usually used for osteomyelitis, septic arthritis or endocarditis can be recommended and then, if *K. kingae* etiology will be confirmed, antimicrobial therapy can be optimized according to the results of the antimicrobial susceptibility test if available or using a second- or third-generation cephalosporin.

A still unsolved problem is how to manage outbreaks of *K. kingae* infection, but it has been suggested that prophylactic antibacterial drugs can be administered to prevent further cases of disease. As it has been found that rifampin, which is particularly active against *K. kingae*, is secreted in saliva and reaches high concentrations in the upper respiratory tract mucosa, and has been effective in eradicating other invasive pharyngeal colonisers such as *Haemophilus influenzae* type b and *Neisseria meningitidis*, it has been suggested that it should be given at a dose of 10 mg/kg twice daily for two days, alone or in combination with amoxicillin 80 mg/kg/day for two or four days [1, 21]. However, the systematic use of rifampin prophylaxis remains a subject of debate because the results have not always been satisfactory as eradication of *K. kingae* was achieved in only some of the treated children or new colonisation by the same strain was observed some weeks later [22]. This was not because of bacterial resistance, but appeared at least partially due to poor compliance. Furthermore, the use of antibiotic prophylaxis is supported by the fact that no further cases of invasive *K. kingae* disease were diagnosed in the affected day care centres even when a few children continue to be colonised by the invasive *K. kingae* strain [22]. Nevertheless, detecting an outbreak of *K. kingae* infection is still difficult, mainly because of the low rate of *K. kingae* testing. It has been suggested that an algorithm for investigating and managing clusters of invasive *K. kingae* infections in day care centres would be useful [9], but the many controversial areas of intervention and the inclusion of genetic analyses to guide management make its use particularly difficult in geographic areas where appropriate laboratory facilities are not available.

## Conclusions

Modern laboratory techniques have led to the identification of *K. kingae* as a significantly more important infectious agent than previously thought. It is a common coloniser of the oropharynx, can be transmitted from child to child, and can cause outbreaks in day care facilities. Invasive infections almost exclusively occur in children aged between six months and four years, and mainly involve joints and bone, less frequently the endocardium, and very rarely other localisations. With the exception of endocarditis, which can be followed by severe complications, the diseases due to *K. kingae* are usually accompanied by mild to moderate clinical signs and symptoms, and only slightly altered laboratory data. Moreover, they generally respond to widely used antibiotics, although resistant strains are reported. However, the mild symptoms and limited increase in the levels of acute phase reactants create problems because *K. kingae* disease may be confused with other clinical conditions that have a similar clinical picture.

It is for these reasons that, although *K. kingae* was identified more than 50 years ago, it is poorly known by pediatricians and is not systematically sought in laboratories. Education is therefore necessary in order to reduce the risk of outbreaks, permit the early identification of *K. kingae* infections, and allow the prompt prescription of adequate therapeutic regimens capable of avoiding the risk of a negative evolution in those cases in which this elusive pathogen can cause significant clinical problems.

## Abbreviations

CRP: C reactive protein
ESR: Erythrocyte sedimentation rate
*Kk*: *Kingella kingae*
PCR: Polymerase chain reaction
WBC: White blood cell count
